# A Transcriptome-Wide Isoform Landscape of Melanocytic Nevi and Primary Melanomas Identifies Gene Isoforms Associated with Malignancy

**DOI:** 10.3390/ijms22137165

**Published:** 2021-07-02

**Authors:** Siras Hakobyan, Henry Loeffler-Wirth, Arsen Arakelyan, Hans Binder, Manfred Kunz

**Affiliations:** 1 Institute of Molecular Biology NAS RA, Yerevan 0014, Armenia; s_hakobyan@mb.sci.am (S.H.); aarakelyan@sci.am (A.A.); 2 Interdisciplinary Centre for Bioinformatics, University of Leipzig, Härtelstr. 16–18, 04107 Leipzig, Germany; wirth@izbi.uni-leipzig.de (H.L.-W.); binder@izbi.uni-leipzig.de (H.B.); 3 Department of Dermatology, Venereology and Allergology, University of Leipzig Medical Center, Philipp-Rosenthal-Str. 23, 04103 Leipzig, Germany

**Keywords:** melanoma, splicing, transcriptome, tumor progression

## Abstract

Genetic splice variants have become of central interest in recent years, as they play an important role in different cancers. Little is known about splice variants in melanoma. Here, we analyzed a genome-wide transcriptomic dataset of benign melanocytic nevi and primary melanomas (*n* = 80) for the expression of specific splice variants. Using kallisto, a map for differentially expressed splice variants in melanoma vs. benign melanocytic nevi was generated. Among the top genes with differentially expressed splice variants were Ras-related in brain 6B (*RAB6B*), a member of the RAS family of GTPases, Macrophage Scavenger Receptor 1 (*MSR1*), Collagen Type XI Alpha 2 Chain (*COLL11A2*), and LY6/PLAUR Domain Containing 1 (*LYPD1*). The Gene Ontology terms of differentially expressed splice variants showed no enrichment for functional gene sets of melanoma vs. nevus lesions, but between type 1 (pigmentation type) and type 2 (immune response type) melanocytic lesions. A number of genes such as Checkpoint Kinase 1 (*CHEK1*) showed an association of mutational patterns and occurrence of splice variants in melanoma. Moreover, mutations in genes of the splicing machinery were common in both benign nevi and melanomas, suggesting a common mechanism starting early in melanoma development. Mutations in some of these genes of the splicing machinery, such as Serine and Arginine Rich Splicing Factor A3 and B3 (*SF3A3*, *SF3B3*), were significantly enriched in melanomas as compared to benign nevi. Taken together, a map of splice variants in melanoma is presented that shows a multitude of differentially expressed splice genes between benign nevi and primary melanomas. The underlying mechanisms may involve mutations in genes of the splicing machinery.

## 1. Introduction

Melanoma is an important cancer entity with an increasing incidence and bad prognosis in the metastatic stage [1]. More recent treatment modalities including targeted treatment and checkpoint inhibitor treatment addressing CTLA-4 and PD-1 signaling in immune cells have improved the overall survival of melanoma patients [1,2]. However, the low response rates of checkpoint inhibitors and high recurrence rates of targeted treatment require a more complete understanding of melanoma biology.

For this purpose, gene expression profiles of melanoma samples at different stages have been analyzed in a series of large-scale transcriptomic studies measuring gene expression. An earlier report used gene expression profiles of thick primary melanomas to predict the overall survival of patients [3]. A 254-gene signature was identified that distinguished between bad and good prognosis groups. Among the top classifying genes were genes involved in cell cycle control, mitosis, and DNA replication. The vast majority of these genes were under-expressed in the good prognosis group. Among genes whose expression was higher in the good prognosis group were immune-related genes. 

A later study showed that gene patterns identified in metastatic lesions may also be of prognostic significance for primary melanomas [4]. While the high-immune response subtype was associated with a better overall survival of patients, the proliferative subtype, characterized by an elevated expression of cell cycle-associated genes, was associated with worse prognosis. In a follow-up study, the same group showed that gene patterns identified in metastatic lesions may have prognostic significance in primary melanomas [5]. Gene Ontology (GO) categories of immune response, DNA repair, and cell cycle were biologic processes that differed between both high-grade (bad prognosis) and low-grade (good prognosis) primary lesions [5]. 

In more recent meta-analyses of several gene expression studies using microarray technology, differentially expressed genes between primary melanomas and melanoma metastases were found to be indicative of a good or bad prognosis [6,7]. In one of these studies, gene patterns were even able to predict treatment response to targeted (BRAF inhibitor) treatment [7]. 

In the most comprehensive study at present, analyzing gene expression and genetic patterns of melanoma samples using RNA-seq technology, three major tumor subtypes were identified, termed “immune”, “keratin”, and “MITF-low”, standing for activated functional gene signatures in these samples [8]. In this study, immune cell cluster patients had the best prognosis. Another more recent study also showed that immune cell characteristics had a strong and positive predictive value regarding treatment response to immune checkpoint therapies in melanoma [9]. In an independent study on melanoma biopsies before treatment, a highly significant correlation of CD4, CD8, CD3, PD-1, and LAG-3 expression in tissue lesions with anti-PD-1 treatment response was observed [10]. Upregulated genes in responders involved HLA molecules, chemokines, and genes of the IFN-γ pathway [10]. Thus, immune gene signatures may have a predictive value for treatment response to immune checkpoint inhibition.

In our recent study using laser-microdissected material and RNA-seq technology, two transcriptomic types of primary melanomas (M1 and M2) were identified [11]. While the M1 type of melanoma lesions (pigmentation type) was characterized by gene signatures of pigmentation and MITF activity and an enrichment of *NRAS*-mutant melanomas, the M2-type lesions (immune response type) showed gene signatures of inflammation, immune genes, and AXL kinase activity, with a low prevalence of *NRAS*-mutant melanomas. We also showed that samples of an independent melanoma cohort could be classified as pigmentation or immune response types [8]. Thus, a large number of gene expression studies performed in melanoma may help to differentiate between benign and malignant lesions, and gene patterns may have a prognostic value. Overall, signatures of the cell cycle and inflammation including cytokines and chemokines appear to be central for the progression of benign melanocytic lesions to melanomas. 

More recent developments showed that gene expression signatures are only part of a more complex picture of differentially expressed genes, and different splice variants may provide a more detailed picture of the underlying biological processes [12,13,14,15]. In particular, alternative splicing of genes is dysregulated in many cancers, but the underlying mechanisms and biological consequences have not yet been analyzed in detail [12,15,16]. Different splice variants of the same gene may even have different functions. Only a few studies on alternative splicing in melanomas have been published thus far. They aimed at identifying spliced genes with a prognostic impact in skin and uveal melanoma [17,18], focused on the splicing of genes encoding RNA-binding proteins [19], or investigated splicing of the immunopeptidome [20].

Here, we performed a large-scale analysis of splice variants that might be of relevance for early melanoma development and that may even divide different subtypes of melanomas. We were particularly interested in the identification of genes differently spliced between benign melanocytic nevi and primary melanomas and between pigmentation and immune response subtypes, as well as associations between splicing and the mutational load of the respective genes and genes of the RNA splicing machinery.

## 2. Results

### 2.1. The Transcriptional and Isoform Landscape of Primary Melanomas

**Figure 1:**
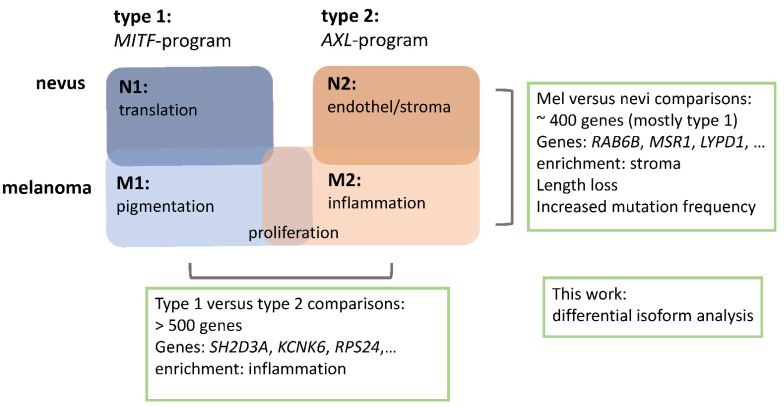
Stratification of the transcriptomes of benign nevi and melanomas into two types as described previously [11]. The functional context of the different subtypes is provided in the boxes. Isoform analyses performed in this work are noted in the boxes with green borders.

Recently, we performed a differential gene expression analysis of 80 melanomas and benign melanocytic nevi [11]. We found that both types of lesions could be separated by two types of gene expression patterns (Figure 1) [11]. Type 1 nevi (N1) showed signatures of genes involved in translation and ribosome activity, while type 1 melanomas (M1) showed strong signatures of pigmentation-related genes. Among the genes involved in these processes were *MITF*, *BRAF*, *CDKN2A*, *YAP1*, *ARID1A*, *MC1R*, and *SOX10*. In contrast, N2 nevi were enriched for signatures involved in endothelial- and stroma-specific genes, and M2-type melanomas were enriched for immune response signatures (*AXL*, *KIT*, *EGFR*, *SMARCA2*, *PDCD1* (PD1), and *CTLA4*). Both types of melanomas showed an increased expression of gene signatures of cell proliferation [11]. This analysis provided the basis for further splice variant analyses in the present study. 

### 2.2. Isoform Switches

#### 2.2.1. Melanomas vs. Nevi

Using the kallisto, DEXseq, and IsoformSwitchAnalyzeR programs, we quantified gene isoform expression in this dataset and compared splice variant expression in melanomas with that in benign melanocytic nevi in order to find variants associated with malignant transformation, similar to previous studies [21,22,23]. The isoform switch identification algorithm is based on the assumption of an antagonistic differential expression of isoforms, meaning that up- or downregulation of one isoform should be paralleled by an expression change in one or more isoforms in the opposite direction. 

**Figure 2:**
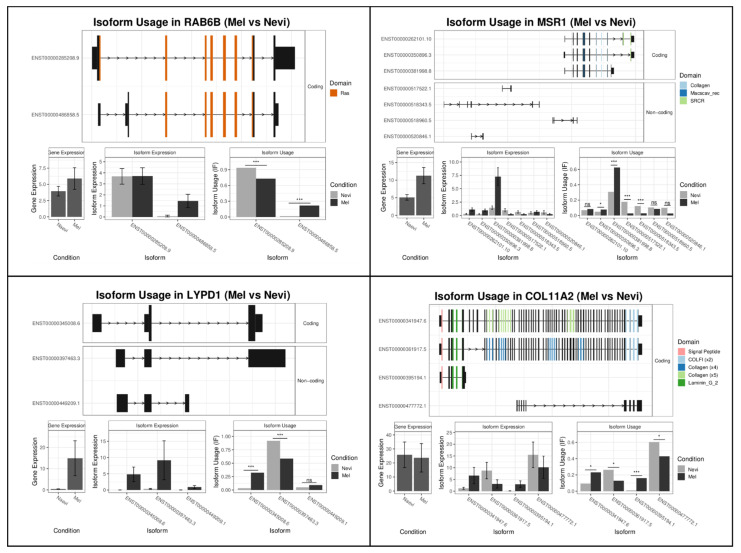
Top spliced genes in melanoma as compared to benign melanocytic nevi. A set of 80 melanoma and benign nevus samples was analyzed by RNA-seq technology after laser microdissection of melanocytic cells. Differential gene expression analysis was performed as described in the Materials and Methods section. For the identification of differentially expressed splice variants, the *kallisto* program was used. Splice variants of 4 from the top 10 spliced genes are shown. SRCR, Scavenger receptor cysteine-rich protein domain; Macscav_rec, Macrophage scavenger receptor. For each gene, the structures of the switched isoforms are shown, as well as gene and isoform expression and usage. *, *p* < 0.05; ***, *p* < 0.001; n.s., not significant.

A large number of isoforms clearly differed between benign melanocytic nevi and primary melanomas and type 1 and type 2 lesions (Table 1, Tables S1 and S2) [11]. Among the top genes with differentially spliced isoforms between primary melanomas and benign melanocytic nevi were *RAB6B*, *MSR1*, *LYPD1*, and *COL11A2* (Table 1; Figure 2). *RAB6B* is a member of the RAS family of GTPases. Little is known about its role in cancer biology, but evidence has been provided that it plays a role in micro-RNA-regulated growth of gastric cancer cells [24]. The most prominent isoform expression effect in melanoma was upregulation of the ENST00000486858.5 isoform characterized by an intron loss. This intron loss presumably leads to functional activation. Thus, this *RAB6B* isoform in melanomas may exert pro-tumorigenic functions. 

Another example of differentially expressed isoforms appeared in the *MSR1* gene (Table 1; Figure 2). The most obvious differences in isoform expression were observed for ENST00000381998.8. This isoform, upregulated in melanoma, is characterized by a gain of collagen and macrophage scavenger receptor domains. The most prevalent *MSR1* isoform in benign nevi was ENST00000517522.1, a non-coding transcript associated with a complete loss of an open reading frame (ORF). MSR1 is a macrophage-specific trimeric integral membrane glycoprotein which is involved in the endocytosis of low-density lipoproteins [25]. Evidence has been provided showing that sequence variants of the *MSR1* gene are associated with short overall survival in pancreatic cancer patients [26,27]. *MSR1* is expressed by type 2 tumor-associated macrophages and may thereby promote tumorigenesis [27,28]. 

*LYPD1* is a modulator of the nicotinic acetylcholine receptor activity. The most obvious differences in isoform fractions were observed for ENST00000345008.6. This isoform, upregulated in melanomas, is a coding transcript with a domain gain. Evidence has been provided showing that *LYPD1* is involved in the pathogenesis of ovarian cancer, and, recently, anti-LYPD1/CD3 bispecific antibodies have been tested for the treatment of this tumor type [29]. 

Among the top differentially expressed splice variants were isoforms of the *COL11A2* gene. *COL11A2* encodes one of the two alpha chains of type XI collagen. Type XI collagen is a heterotrimer where the third alpha chain is post-translationally modified. It interacts with COL5A1, which was also among the top differentially expressed splice variants (Supplementary Table S1). Isoforms upregulated in melanoma were ENST00000341947.6 and ENST00000395194.1, characterized by domain gain and domain loss, respectively, but with complete open reading frames. *COL11A2* has been shown to be associated with cancer predisposition in a study of 41 cancer-discordant monozygotic twin pairs [30]. In this study, epigenome-wide analyses identified one novel differentially methylated position in *COL11A2* which might be a mechanism for gene silencing in benign lesions. 

#### 2.2.2. Type 1 vs. Type 2 Lesions

**Figure 3:**
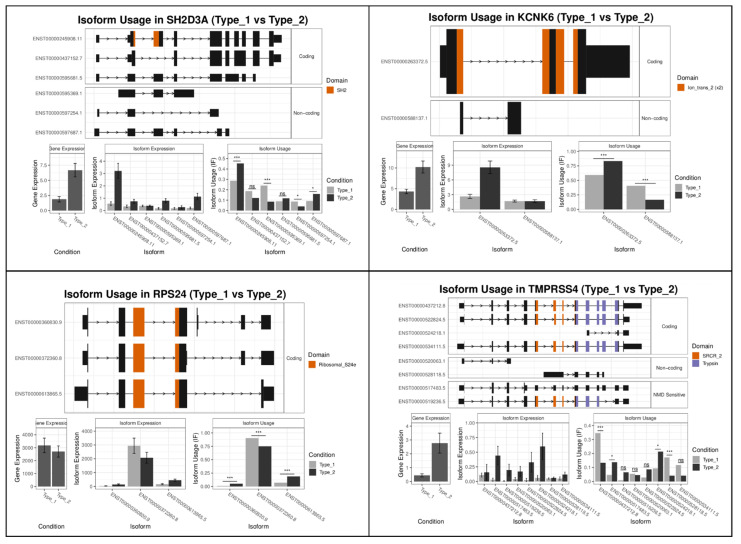
Top spliced genes in type 1 compared to type 2 lesions. A set of 80 melanoma and benign melanocytic nevus samples was analyzed by RNA-seq technology after laser microdissection of malignant cells. Differential gene expression analysis was performed as described in the Materials and Methods section. For the identification of differentially expressed splice variants, the *kallisto* program was used. Splice variants of the four top genes are shown. *, *p* < 0.05; ***, *p* < 0.001, n.s., not significantt.

Next, we evaluated the differences between subtypes of melanocytic lesions, comparing type 1 (translation/pigmentation/MITF) with type 2 (stroma/immune response) phenotypes. Among the differentially expressed isoforms were *SH2D3A*, *KCNK6*, *RPS24*, and *TMPRSS4* (Figure 3). The variant ENST00000245908.11 of SH2 Domain Containing 3A (SH2D3A) was the most significantly upregulated isoform in type 2 lesions. The *SH2D3A* gene encodes for a guanyl nucleotide exchange factor and may thereby interact with RAS or RAC1 signaling. In line with this, the isoform ENST00000245908.11 was highly expressed in type 2 (NRAS-mutant) lesions. It has complete ORF and domain gains compared with the non-coding ENST00000595369.1 isoform. The domain gain refers to an additional SH2 binding domain. SH2D3A also interacts with Neural Precursor Cell Expressed, Developmentally Down-Regulated 9 (*NEDD9*) and Epidermal Growth Factor Receptor (EGFR). A complex of the GTPase RAC1-containing NEDD9 drives mesenchymal-type movement in melanoma cells [31]. EGFR plays a role in melanoma initiation in murine models, while its role in human melanoma is less clear.

The second most significantly dysregulated gene was Potassium Two Pore Domain Channel Subfamily K Member 6 (*KCNK6*). *KCNK6* is a member of the superfamily of potassium channel proteins containing two pore-forming P domains. The most significantly upregulated splice variant ENST00000263372.5 in type 2 lesions has a complete open reading frame and domain gain, in comparison with the non-coding ENST00000588137.1 isoform. The role of *KCNK6* in cancer is not well understood, but under-expression of *KCNK6* and *KCNK15* was associated with the triple-negative subtype of breast cancer [32]. 

The third most significant gene was Ribosomal Protein S24 (*RPS24*). The *RPS24* gene encodes a component of the 40S ribosome subunit and interacts with a number of other ribosomal proteins such as RPS11, 12, and 17. The RPS24c isoform is involved in tumor angiogenesis in colorectal cancer [33]. The most prominent isoform ENST00000613865.5 has lost one of its exons in comparison with ENST00000372360.8 and is highly expressed in type 2 samples. 

The fourth most significant gene in this comparison was Transmembrane Serine Protease 4 (*TMPRSS4*), which encodes a member of the serine protease family with a variety of biological functions. The most prominent isoform ENST00000437212.8, overexpressed in type 1 lesions, had a loss of an ORF and domain loss, arguing for functional inactivation. ENST00000528118.5, also overexpressed in type 1 lesions, has an intron loss, arguing for functional activation. *TMPRSS4* has been shown to be upregulated in various cancers and is regarded as a proto-oncogene. Recently, it could be shown that TMPRSS4 promotes cell proliferation in pancreatic cancer cells and inhibits apoptosis by activating the ERK1/2 pathway [34]. TMPRSS4 induces urokinase-type plasminogen activator (uPA) expression in cancer cells and promotes tumor cell invasion [35].

### 2.3. Functional Categories of Enriched Splice Variants

To further address the question whether individual functional categories were enriched for specific splice variants found in our sample set, we used a number of Gene Ontology (GO) categories. As shown in Table 2 and Table S3, there was no significant enrichment of functional categories in the comparison of melanomas vs. benign melanocytic nevi. The most enriched term was GO:0030198 (extracellular matrix organization; FDR = 0.19). 

In the type 1 vs. type 2 comparison, the category immune response (GO:0006955) showed significant enrichment (FDR = 0.00017), which was also significant for splice variants with presumable functional relevance (FDR = 0.036, Table 2). Table 2 shows an overview on immune-related genes with different splice variant expressions between type 1 and type 2 lesions.

### 2.4. Overlap between Isoform Switches

**Figure 4:**
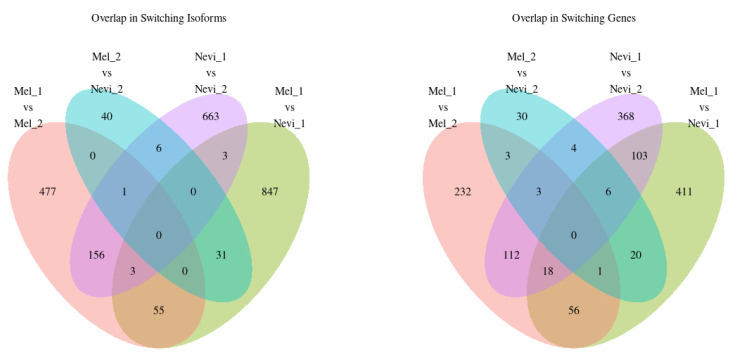
Overlap of splice switching isoforms and splice switching genes. Venn diagrams were generated for differentially expressed splice variants and spliced genes.

Next, we addressed the question whether different subtypes of lesions showed common splice variants. As shown in Figure 4, melanoma type 1 (M1) vs. nevus type 1 (N1) lesions had twice the number of specifically differentially expressed splice variants (*n* = 847) compared with M1 vs. M2 (*n* = 477), with only a small overlap between both comparisons (*n* = 55). This result suggests that the majority of variants were associated with malignant transformation of type 1. Note that, in contrast, only *n* = 40 variants were specifically different between M2 and N2, indicating that splicing seems to have only a relatively small effect on malignant transformation of type 2 lesions. The difference in splicing between type 1 and type 2 is most prominent in benign nevi (*n* = 663 for N1 vs. N2). In line with this, *n* = 156 of the variants present in the N1 against N2 comparison were also present in the M1 vs. M2 comparison, which is suggestive for signatures that differ between both types of lesions. Similar relations were found for the number of switched genes, but their number is roughly reduced by a factor of two, meaning that, on average, each gene is represented by two to three transcript isoforms. Overall, the vast majority of splice variants and genes were characteristic for a specific subtype.

### 2.5. Categories of Isoform Switching

**Figure 5:**
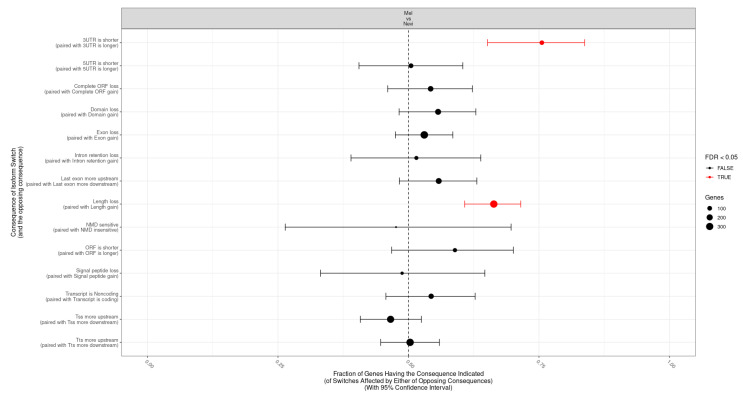
Transcriptomic consequences of isoform switches between melanoma and nevus samples. The fraction of genes (x axis) with indicated consequences (y axis) when comparing melanomas to benign melanocytic nevi is shown.

Next, the most prominent categories of transcriptomic regions affected by splice variants were analyzed. As shown in Figure 5, when comparing melanomas with nevi, significant enrichment was observed for a shortened 3′ untranslated region (3′UTR) in melanomas. Since the 3′UTR regions contain binding motifs for small interfering RNAs (e.g., microRNAs), shortening of this region might be associated with deregulated gene expression. Indeed, a shortened 3′UTR is a common finding in cancer cells [36,37]. Moreover, there was an overall shortening of the gene length (Figure 5). In the analysis of type 1 vs. type 2 lesions, a loss of an ORF and a domain loss were observed, arguing for a predominance of functionally inactivated genes (Figure S1). This was also observed when comparing M1 vs. M2 lesions and N1 vs. N2 lesions. Taken together, categories of isoform switching were associated with functional gene regions.

### 2.6. Mutation Analysis

Differential splicing might be due to genetic alterations, e.g., in upstream polymorphic regions, or in splice acceptor or splice donor sites. In order to address this issue, we analyzed sequence variants in our sample set. We were particularly interested in mutations present in splice sites of spliced genes or overall mutations in spliceosomal genes, and whether these were associated with individual splice variants. First, we tested whether genes with switched isoforms in melanoma samples had a higher proportion of mutations compared with these genes in nevus samples. For each gene, a Z-score statistic was calculated by comparing the mutation count difference of spliced genes between melanomas and nevi with the mutation count difference of selected 100 genes with a similar median isoform length. Then, genes exceeding the 90% confidence interval were selected as having a higher prevalence of mutations in melanomas. 

**Figure 6:**
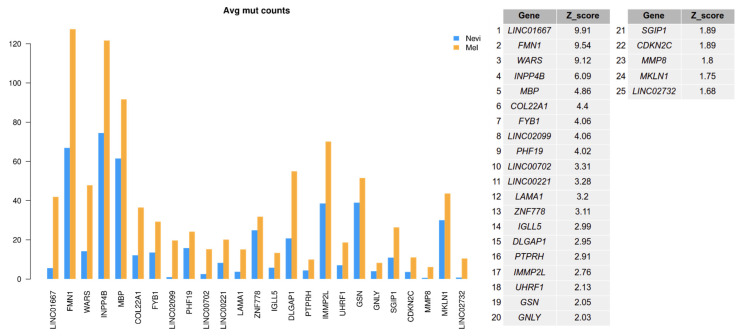
Number of mutations by gene in melanomas compared to benign melanocytic nevi. A mutation analysis was performed from the basic set of samples sequenced by RNA-seq. Only mutated genes of switched isoforms above a significance score (Z-score) of 1.6 with a 90% confidence interval are shown.

**Figure 7:**
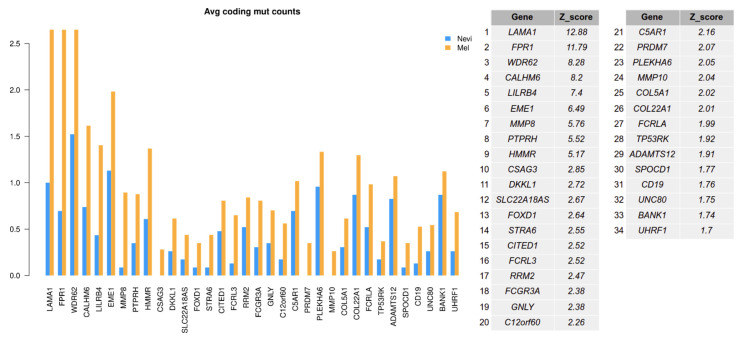
Number of coding mutations by gene in melanomas compared to benign melanocytic nevi. A mutation analysis was performed from the basic set of samples sequenced by RNA-seq for coding regions of genes. Only mutated genes of switched isoforms above a significance score (Z-score) of 1.6 with a 90% confidence interval are shown.

As shown in Figure 6 (all mutations) and Figure 7 (mutations in coding genes), there was an enhanced mutation rate in melanomas as compared to benign nevi for a number of genes which presented with an isoform switch. Among these genes were *FMN1*, *COL22A1*, *LAMA1*, and *MMP8*. Matrix Metallopeptidase 8 (*MMP8*) had also been described in an earlier mutational analysis in melanoma [38]. Formin 1 (*FMN1*) is involved in the formation of adherens junctions and actin organization, and other formin family genes (*FMNL2/FMNL3*) have been shown to play a role in melanoma biology [39]. Different formins contribute to the velocity of lamellipodium protrusions and thereby to the cell migration of melanoma cells [40].

Laminin Subunit Alpha 1 (*LAMA1*) was detected as the top gene on the list for spliced genes with coding mutations. *LAMA1* encodes the alpha 1 subunit of laminin. Laminins are extracellular matrix glycoproteins that make up a major component of the basement membrane and have been implicated in cell adhesion, migration, signaling, and tumor metastasis [41,42]. Another gene, Formyl Peptide Receptor 1 (*FPR1*), is normally expressed by phagocytic cells and is involved in host defense and inflammation. This protein has also been shown to be involved in melanoma cell migration [43]. Thus, mutated genes found in this analysis may have an impact on melanoma biology, affecting cell migration.

**Figure 8:**
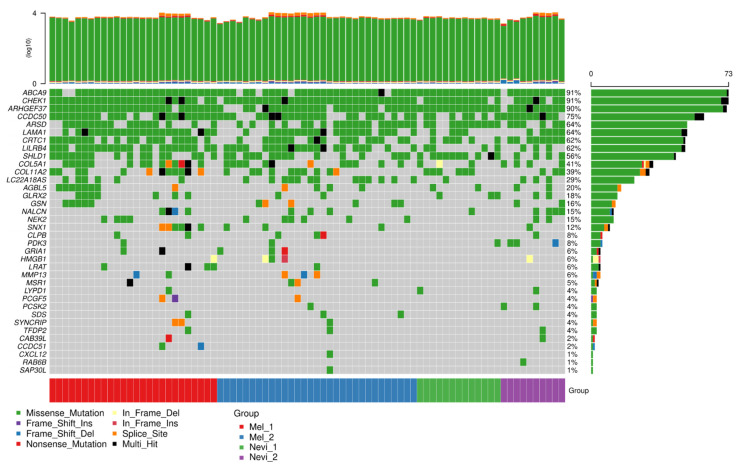
Mutational load of spliced genes in the comparison of primary melanomas against benign melanocytic nevi. A mutation analysis was performed from the basic set of samples sequenced by RNA-seq for coding regions of genes. Shown are the top spliced genes in the present study.

Next, we asked whether the top spliced genes differ in their mutational load as a possible explanation for differential splicing. As shown in Figure 8, top spliced genes were mutated in a large proportion of samples, including benign melanocytic nevi and melanomas. Interestingly, these mutations were homogeneously distributed among both entities. This result suggests that benign melanocytic nevi already carry mutations of genes that are subjected to differential splicing in melanoma, and that the mutational load alone may not explain differences in the usage of splice variants. Among the top mutated genes were *ABCAP*, *CHEK1*, *CCDC50*, *ARSO*, and *LAMA1*, which showed mutations in more than 60% of the samples. The vast majority were missense mutations, while splice site mutations were less prominent, arguing against a significant role of splice site mutations in melanoma splicing events. 

ATP Binding Cassette Subfamily A Member 9 (*ABCA9*) showed the highest prevalence for mutations. ABC proteins such as ABCA9 transport various molecules across the extra- and intracellular membranes and thereby contribute to tissue homeostasis. They accumulate in subfractions of cells in melanoma short-term cultures, as shown recently [44]. *CHEK1* is a major DNA repair checkpoint molecule and is involved in the pathogenesis of a variety of cancers, arguing for a role of failed DNA repair in these malignancies [45]. Coiled-Coil Domain Containing 50 (*CCDC50*) encodes a soluble, cytoplasmic, tyrosine-phosphorylated protein with multiple ubiquitin-interacting domains. It also has a function as a negative regulator of NF-kB signaling and is an effector of EGF-mediated signaling. Taken together, spliced genes harbor a plethora of mutations which might contribute to the process of splicing and also functional inactivation of these genes during tumor progression.

**Figure 9:**
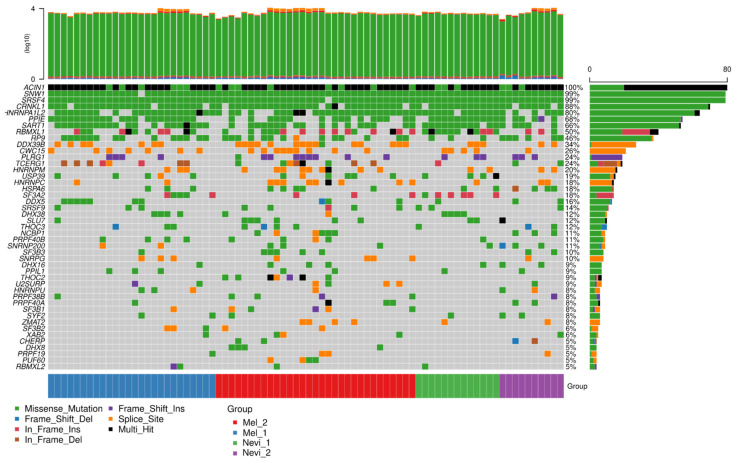
Mutational load of spliceosomal genes in the comparison of primary melanomas with benign melanocytic nevi. Mutational load of spliceosomal genes as annotated in the KEGG list at https://www.genome.jp/kegg-bin/show_pathway?ko03040, accessed on 4 December 2018.

**Figure 10:**
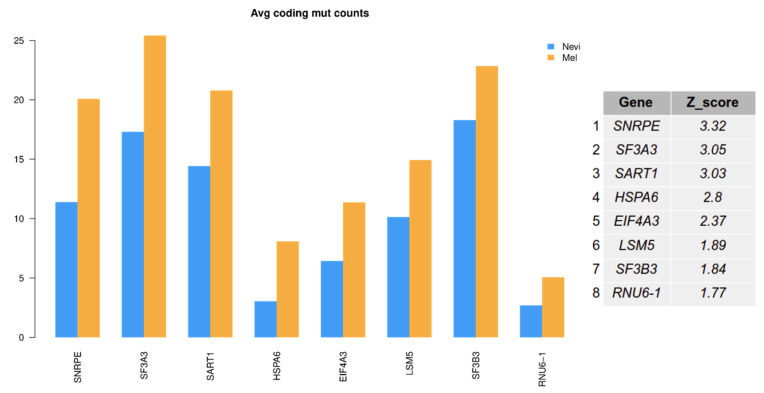
Significance score of mutational load of spliceosomal genes in the comparison of primary melanomas with benign melanocytic nevi. The plot shows spliceosome genes with significant average mutation count difference. Z-score was estimated by constructing a distribution of 200 randomly selected switched isoforms of similar length.

In a further analysis, it was tested whether genes of the splicing machinery showed an enhanced mutational load in melanomas as compared to benign melanocytic nevi, using the KEGG annotations of spliceosomal genes (https://www.genome.jp/kegg-bin/show_pathway?ko03040; accessed on 4 December 2018). All samples showed at least one gene of the splicing machinery to be mutated (Figure 9). The most prominent genes were *ACIN1*, *SNW1*, *SRSF4*, *CRNKL1*, *HNRNPA1L2*, *PPIE*, and *SART1*, with high mutational rates in more than 60% of the samples. Apoptotic Chromatin Condensation Inducer 1 (*ACIN1*) is part of a splicing-dependent multiprotein exon junction complex located at splice junctions on mRNAs. It also induces apoptotic chromatin condensation. SNW Domain Containing 1 (*SNW1*) is involved in splicing by interacting with poly(A)-binding protein 2. Serine And Arginine Rich Splicing Factor 4 (*SRSF4*) is a member of the serine/arginine-rich splicing factor family [46]. Another member of this family, SRSF3, has recently been discussed as a target for cancer therapy [47]. There was also a significant correlation for a number of spliceosomal genes with an enhanced mutational load in melanomas (Figure 10). 

To estimate the association between mutations and isoform switches, we calculated the Jaccard index (J index) for the isoforms of the top 50 significantly switched genes in the melanoma vs. benign melanocytic nevus comparison. For each isoform, the J index is calculated as a ratio of the number of melanoma samples which have both a switch and a mutation(s) (intersection) divided by the number of samples which have a switch and/or a mutation (union) in that particular isoform. Then, the fraction of mutated samples is plotted as a function of the J index, estimating the fraction of switched and mutated samples. Strong associations refer to large values of both fractions.

**Figure 11:**
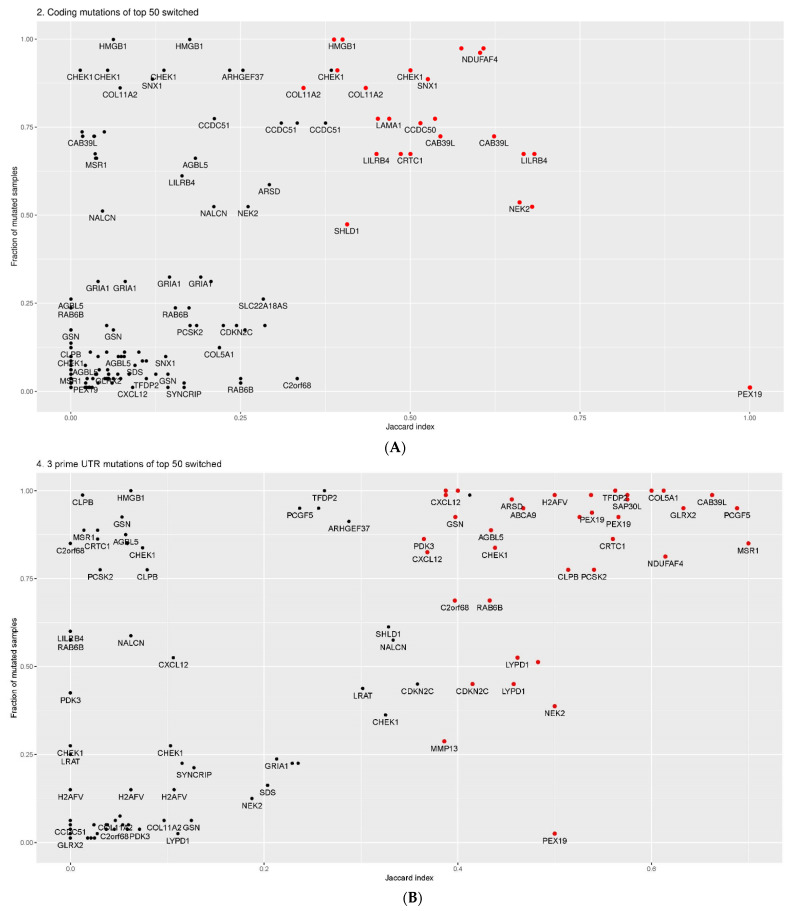
Jaccard index plots for the association of isoform switches with mutations. (**A**) Jaccard index (x axis) for coding mutations with switched isoforms of top 50 switched genes against frequency of mutated samples (y axis). (**B**) Jaccard index plot for mutations located in 3′UTR. Z-score for calculated J index was estimated by constructing a distribution of Jaccard indices of 200 randomly selected switched isoforms of similar length. Z-score by comparing the actual J-value with the J index of 200 different switched isoforms of similar length from melanoma vs. nevus comparison. Each dot corresponds to one isoform and is labeled with its gene name. Significant genes with Z-scores above 1.6 are marked as red (*p* < 0.05).

The analyses showed that coding mutations and 3′UTR mutations were strongly associated with the top switched isoforms where the fraction of mutated samples exceeds the J index in most cases (Figure 11A,B). Overall, the most prominent examples of associations between mutations, splicing, and fraction of samples were observed for *NDUFAF4*, *LILRB4*, *CHEK1*, *SNX1*, *NEK2*, and *CAB39L*, located in the right upper part of Figure 11A. NADH:Ubiquinone Oxidoreductase Complex Assembly Factor 4 (*NDUFAF4*) is involved in the transfer of electrons from NADH to ubiquinone in the mitochondrial respiratory chain. Little is known about its role in cancer, but an earlier report showed that *NDUFAF4*, also termed *HRPAP20*, regulates breast cancer cell invasion [48]. Leukocyte Immunoglobulin Like Receptor B4 (*LILRB4*) is a member of the leukocyte immunoglobulin-like receptor (LIR) family involved in inhibition of stimulation of an immune response. The particular role of *CHEK1* in DNA repair and cancer biology has been mentioned above. Sorting Nexin 1 (*SNX1*) is an endosomal protein and regulates the cell surface expression of EGFR. NIMA Related Kinase 2 (*NEK2*) is a serine/threonine protein kinase that is involved in mitotic cell regulation. NEK2 expression is associated with early relapse and poor prognosis in a number of different cancers [49]. 

Calcium Binding Protein 39 Like (*CAB39L*) is related to G protein-coupled receptors (GPCR) and Rearranged during Transfection (RET) signaling. Taken together, significant associations between mutational patterns, splicing, and frequency of occurrence in samples were observed in genes with relevance for cancer development. This argues against a mere random correlation based on the known high mutation frequency in melanomas. The association with 3′UTR regions of a number of genes supports the notion that this region has an important impact on splicing.

## 3. Discussion

Aberrant gene splicing is a common finding in many cancers [12,15,16]. Splicing mechanisms include co-transcriptional RNA processing such as RNA Pol II carboxy-terminal domain-mediated recruitment of splicing factors, kinetic competition between these factors, and the influence of the chromatin structure [42]. Kinetic competition influences the use of weak or strong splice sites, and the chromatin structure influences splice site choices. Different splice variants are associated with primary tumors and metastases when compared to normal tissue. Classical examples are E-cadherin, CD44, TP53, and EGFR [50,51]. However, there is little overlap between individual splice variants and different cancer types [16]. 

Based on an earlier RNA-seq melanoma study, we analyzed splice variants in a set of 80 melanoma and benign melanocytic nevus samples [11]. Here, we used the kallisto program to identify splice variants associated with malignant transformation as well with specific mutational patterns of spliced genes and mutational patterns of genes of the splicing machinery. We provide a resource of melanoma splice variants that may be used for future functional analyses. A set of highly differentially expressed splice variants was found (close to 400) when comparing benign lesions (melanocytic nevi) with malignant lesions (melanomas). Even more striking, there were more than 700 differentially expressed splice variants when comparing type 1 (translation/pigmentation) lesions and type 2 (stroma/immune response) lesions, which shows that the two different backgrounds of melanoma development are associated with different splicing activities. As described earlier, type 1 (N1/M1) lesions are characterized by pigmentation-type and MITF gene signatures and prevalent NRAS mutations in M1 melanomas [11]. These lesions are associated with enhanced splicing. Type 2 (N2/M2) lesions were characterized by inflammatory-type and AXL gene signatures, with remarkably less splicing events [11].

Among differentially expressed splice variants in the comparison of melanomas with benign melanocytic nevi, *RAB6B*, a member of the RAS oncogene family, was the top gene with differentially expressed spliced variants between benign and malignant lesions. Evidence for a role of *RAB6B* in cancer development is scarce, but the gene has been shown to be involved in GTP binding and may function in the retrograde Golgi transport in neuronal cells [52]. RAB6B interacts with RAB1 and RAB2, two other family members, both involved in intracellular signaling. In gastric cancer cells, miR-4268 expression negatively correlated with Rab6B expression, and miR-4268 overexpression repressed gastric cancer cell growth [24]. The effects were mediated via AKT/JNK signaling pathways. The dominant variant found in melanoma was characterized by an intron loss, which may lead to reactivation of the *RAB6B* gene and may thereby exert pro-tumorigenic functions.

Splice variants in GTP-binding proteins might be used for innovative treatment approaches in cancer. In a recent report on splice switching in breast cancer biopsies, two different isoforms of Rap1 GTPase-GDP Dissociation Stimulator 1 (*RAP1GDS1*) (protein name SmgGDS) were identified, called SmgGDS-607 and SmgGDS-558, both involved in prenylation of multiple small GTPases that contain a C-terminal polybasic region, including Rac1, RhoA, Kras, and Rap1 [53]. The authors identified an enhanced ratio of splice variants in cancer against normal tissues, which was associated with reduced survival of breast cancer patients. An oligonucleotide inhibiting splice switching was synthesized which reduced the splice variant ratio. This resulted in reduced cancer cell proliferation, inhibited prenylation of a set of small GTPases, and reduced tumorigenesis in genetically modified mice, which normally develop highly metastatic breast cancers [54]. Thus, the small GTPases may serve as targets after activation by spliced effector genes.

A second significantly spliced gene in the present study was *MSR1*. The genetic variant found in melanoma is associated with a domain gain. Sequence variants of the *MSR1* gene are associated with pancreatic cancer, namely, among families affected with hereditary pancreatic cancer, where six rare missense mutations and one nonsense germline mutation were found in *MSR1* [27]. High co-expression of CD44/CD133 and CD204 (MSR1) was associated with short overall survival in another study on pancreatic carcinoma [26]. Moreover, *MSR1* is expressed by M2-type tumor-associated macrophages and may thereby promote tumorigenesis [28]. Mutations were present in all functional domains (e.g., scavenger, collagen-like, and transmembrane domains). 

A recent large-scale study on more than 4000 cancer samples from the Cancer Genome Atlas Project identified 1178 genes with significant isoform changes in at least one cancer type [15]. Of these, 244 isoform switches occurred in the most abundant transcript, which makes them likely to be of functional significance. There was no significant correlation between mutational burden or individual mutations and specific splice variants in most cases. 

Interestingly, we found an association of the number of mutations per gene and the presence of specific splice variants in melanoma, e.g., for mutations in *FMN1*, *COL22A1*, *LAMA1*, and *MMP8*. However, there was no significant association with mutations in splice sites. Even more important, spliceosome genes were mutated in all samples (melanomas and benign melanocytic nevi). Some spliceosome genes showed mutations in up to 80% of the samples. There was an enrichment of mutations in melanoma for a number of genes of the splicing machinery such as Small Nuclear Ribonucleoprotein Polypeptide E (*SNRPE*), *SF3A3*, and *SF3B3*. The *SNRPE*-encoded protein is a core component of U small nuclear ribonucleoproteins, which are key components of the pre-mRNA processing spliceosome. It also plays a role in the 3’ end processing of histone transcripts. *SF3A3* encodes subunit 3 of the splicing factor 3a protein complex, and *SF3B3* encodes subunit 3 of the splicing factor 3b protein complex. Both complexes play an important role in splicing, chromatin modification, transcription, and DNA repair. As recently shown, spliceosome-mutant cancers might be particularly sensitive to H3B-8800, a particular inhibitor of this structure, which might open future perspectives for melanoma treatment [54].

Among the genes with splice variants described in the study by Sebestyén and co-workers were a number of genes with oncogenic potential, among them *CCND3* and *CDKN2C* [15]. *CCND3* and *CDKN2C* were also shown to be alternatively spliced in the present study. We found an increased expression of a *CCND3* variant with a domain gain in the N-terminal region in melanomas. *CCND3* variants have also been described in breast cancer [15], but their functional relevance remains to be determined. 

Alternative splicing may affect tumor progression towards metastasis [50]. Among top differentially expressed gene variants in metastatic lesions were genes coding for CEA Cell Adhesion Molecule 1 (*CEACAM1*), tropomyosin 1 (*TPM1*), and several chemokines and chemokine receptors such as C-X-C Motif Chemokine Ligand 12 (*CXCL12*) and C-X-C Motif Chemokine Receptor 3 (*CXCR3*). *CEACAM6* and *CEACAM19*, but not *CEACAM1*, were also differentially spliced in type 1 vs. type 2 lesions in the present study. *TPM1* and *CXCL12* were alternatively spliced in the melanoma vs. nevus comparison. *TPM1* had a longer 5′ UTR region in melanomas. TPM1 is an actin-binding protein that stabilizes actin filaments and affects integrin expression. It has a potential role as a tumor suppressor, as it is downregulated in a number of cancers such as bladder, breast, and colorectal cancer, and disrupted cytoskeletal structures may promote malignancy [55]. The *CXCL12* splice variant in melanoma had an intron loss, which argues for a functional activation. The *CXCL12* protein, also termed SDF-1, has six alternatively spliced variants, and the SDF-1β and SDF-1γ variants are associated with tumor size in colorectal cancer [56].

Taken together, melanomas and nevi differ in their expression of a high number of genes with individual splice variants. However, the functional relevance for many of these variants remains to be determined. Interestingly, melanomas show a significantly higher mutational burden than benign melanocytic nevi in several spliceosome genes, which might, at least in part, explain the high number of alternative splice variants in melanoma and might also have therapeutic consequences. 

## 4. Materials and Methods

### 4.1. Nevi and Melanoma Cases and RNA-Seq Data

Overall, 80 biopsies of primary melanoma samples (*n* = 57) and benign melanocytic nevi (*n* = 23) were analyzed following informed consent after approval by the local Ethics Committee of the Medical Faculty of the University of Leipzig (Nr. 224–11–11072011; 26 July 2011). All analyses were performed according to the principles expressed in the Declaration of Helsinki. For a more detailed description of the clinical samples and annotations, see our earlier study [11].

### 4.2. Isoform Abundance Quantification Analysis

For isoform-level expression analysis, the kallisto aligner was used [22]. Kallisto provides transcript abundance quantification from RNA-seq data. It uses a novel method of pseudoalignment for fast mapping of reads to reference transcripts with accuracy comparable to conventional aligners [22]. Genecode v32 transcript annotations were used as a reference for the quantification of RNA-seq reads. After abundance quantification, low-expression transcripts were removed. Downstream analysis of isoform switches was performed using the IsoformSwitchAnalyzeR package from R.

### 4.3. Isoform Switches

For the isoform switch analysis, isoform-level transcripts were annotated with gencode v32 and processed with the DEXseq algorithm [23]. DEXseq identifies pairs of isoforms with large opposite changes in isoform usage across conditions where at least one of the changes is statistically significant (FDR-adjusted *p* value (or q-value) < 0.05). Isoform switch identification was performed between two comparison groups: melanomas vs. benign melanocytic nevi and type 1 vs. type 2 lesions. After identification of switched isoforms, the sequences of transcripts were extracted for functional analysis. For this purpose, several tools were used, namely, CPC2 for prediction of coding potential, Pfam (https://www.ebi.ac.uk/Tools/hmmer/search/hmmscan; accessed on 14 October 2019) for domain identification, and SignalP (http://www.cbs.dtu.dk/services/SignalP/, accessed on 4 December 2018) for prediction of signaling peptides in switched isoforms [57,58,59]. 

### 4.4. Mutation Analysis of RNA-Seq Data

Mutation calling from RNA-seq data was performed with the GATK best practice pipeline [60]. RNA-seq reads were aligned to the hg38 reference genome with the STAR 2-pass procedure, and GATK HaplotypeCaller 4.1.4.0 (https://gatk.broadinstitute.org/hc/en-us/sections/360007279452-4-1-4-0, accessed on 28 June 2021) was used for variant calling with default variant filtering options [61]. Identified variants were annotated with the annovar annotator and then visualized with the R maftools package [62,63]. 

## Figures and Tables

**Table 1:** IF switches between top 10 switched isoforms for two comparison groups.

Rank	Gene Name Mel-vs-Nevi	Gene Switch_q_Value	Gene Name Type1-vs-Type2	Gene Switch_q_Value
1	*RAB6B*	2.02 × 10^−14^	*SH2D3A*	2.49 × 10^−19^
2	*MSR1*	1.69 × 10^−12^	*KCNK6*	5.28 × 10^−17^
3	*LYPD1*	3.07 × 10^−12^	*RPS24*	3.40 × 10^−13^
4	*COL11A2*	1.36 × 10^−11^	*TMPRSS4*	3.09 × 10^−12^
5	*GRIA1*	1.06 × 10^−10^	*NEBL*	4.83 × 10^−12^
6	*TFDP2*	1.67 × 10^−10^	*CYSLTR1*	4.83 × 10^−12^
7	*CHEK1*	1.07 × 10^−9^	*PRKCH*	5.34 × 10^−12^
8	*NALCN*	1.94 × 10^−9^	*ICOSLG*	5.89 × 10^−12^
9	*SYNCRIP*	2.86 × 10^−9^	*SMAGP*	6.09 × 10^−12^
10	*C2orf68*	3.57 × 10^−9^	*LFNG*	8.28 × 10^−12^

**Table 2:** Summary of differentially expressed functional splice variants of GO category immune response.

Type 1 vs. Type 2 Filtered (Performed on Subset of Genes Which Had a Functional Isoform Switch)					
Gene Set	Biological Process	Enrichment Ratio	*p* Value	FDR	userId
GO: 0006955	Immune response	1.77	7.30 × 10^−6^	0.036	*AOC1; MMP25; CD6; PRKCH; TNFRSF17; TXK; ICAM3; CEACAM6; HAMP; LFNG; CRTAM; CCR6; ITK; ARG1; LRP1; ZBP1; F12; GCH1; CHI3L1; KLRD1; KLRC1; BLK; IL18BP; MMP7; KLRG1; RHOF; IGSF6; VAV1; PRKACB; FCGR2A; IL2RG; FCRL3; CD300LG; INAVA; PYHIN1; AIM2; DNASE1L3; CLEC4E; NOD2; RAB4B; IL7R; ADORA2B; FPR1; CXCR6; CTSW; KLHL6; CYSLTR1; XCR1; TLR5; CARD9; ZP3; SEMA4A; C5AR1; SPN; MBP; RAET1G; LIME1; PSMB10; CFI; PVRIG*
GO: 0031349	Positive regulation of defense response	2.91	7.94 × 10^−6^	0.036	*CD6; TXK; ICAM3; CRTAM; ARG1; ZBP1; LDLR; F12; VAV1; PRKACB; FCRL3; INAVA; PYHIN1; AIM2; CLEC4E; NOD2; ADORA2B; TLR5; CARD9; ZP3; TGM2; RAET1G*

## Data Availability

The RNA-seq data have been deposited in the NCBI’s Gene Expression Omnibus under GEO: GSE112509.

## Supplementary Materials

The following are available online at https://www.mdpi.com/article/10.3390/ijms22137165/s1.
