# Supplementary material for: A Transcriptome-Wide Isoform Landscape of Melanocytic Nevi and Primary Melanomas Identifies Gene Isoforms Associated with Malignancy
Source: Int J Mol Sci. 2021 Jul 2;22(13):7165. doi: 10.3390/ijms22137165 (PMC8268681; doi:10.3390/ijms22137165)
Supplement: Supplementary file 1 [file ijms-22-07165-s001.zip › 02_Figure_S1_30062021.pdf]

(A)

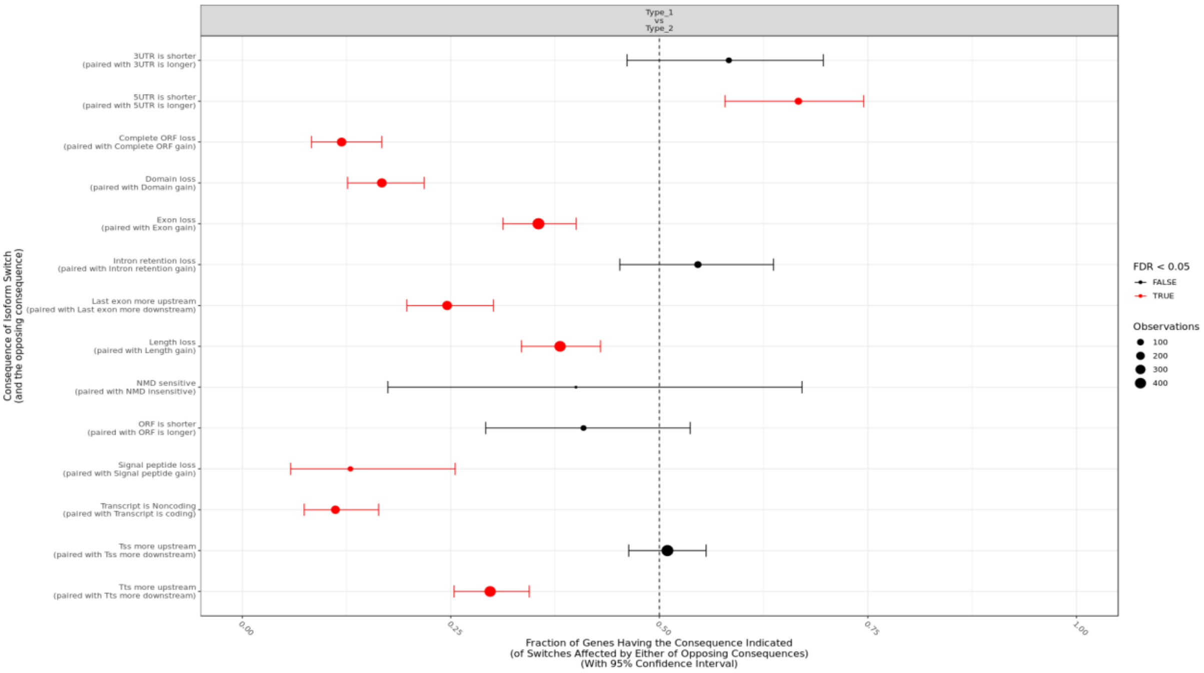

(B)

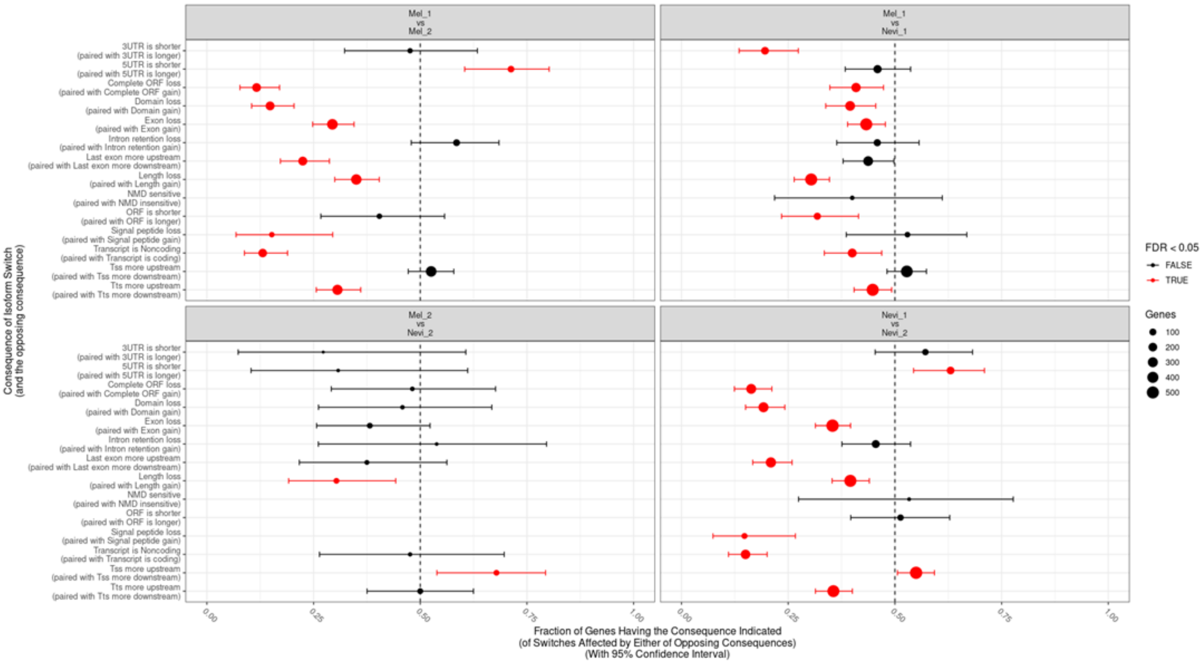

Figure S1A and S1B. Transcriptomic consequences of isoform switches between type 1 and type 2 melanocytic lesions. The fraction of genes (x axis) with indicated consequences (y axis) when comparing type 1 and type 2 lesions is shown.
